# Network and systems biology approaches help investigate gene regulatory interactions between *Salmonella* disease and host in chickens: Model‐based in silico evidence combined with gene expression assays

**DOI:** 10.1002/vms3.70006

**Published:** 2024-10-11

**Authors:** Reza Tohidi, Hoda Javaheri Bargourooshi, Arash Javanmard

**Affiliations:** ^1^ Department of Animal Science Faculty of Agriculture University of Torbat‐e Jam Torbat‐e Jam Iran; ^2^ Department of Animal Production Management Animal Science Research Institute of Iran (ASRI) Agricultural Research Education and Extension Organization (AREEO) Karaj Iran; ^3^ Department of Animal Science Faculty of Agriculture University of Tabriz Tabriz Iran

**Keywords:** candidate gene, genetic resistance, polymorphism, *Salmonella* enteritidis

## Abstract

**Background:**

*Salmonella enteritidis* (SE), a previously widespread infectious disease, is still cited as a major factor in economic losses in commercial chicken production. The host's genetic immune system determines the pathogenicity of a particular bacterium. To shed light on this topic, it was necessary to understand the key candidate genes essential for regulating susceptibility and resistance to the target disease. The field of poultry farming in particular has benefited greatly from the connection between quantitative and molecular genetics.

**Objectives:**

This study aims to identify the most important immune‐related genes and their signalling pathways (gene ontology, co‐expression and interactions) and to analyse their accumulation in host‐resistant SE diseases by combining gene expression assays with model‐based in silico evidence.

**Methods:**

A two‐step experimental design is followed. To start, we used free computational tools and online bioinformatics resources, including predicting gene function using a multiple association network integration algorithm (geneMania), the Kyoto Encyclopedia of Genes and Genomes, the Annotation, Visualization and Integrated Discovery (DAVID) database and the stimulator of interferon genes. Natural resistance‐associated macrophage protein 1 (*NRAMP1*), Toll‐like receptor 4 (*TLR4*), interferon‐γ (*IFNγ*), immunoglobulin Y (*IgY*) and interleukin 8 (*IL8*) were among the five genes whose expression levels in liver, spleen, and cecum were evaluated at 1107 SE after 48 h of inoculation. This molecular study was developed in the second phase of research to validate the in silico observations. Next, we use five promising biomarkers for relative real‐time polymerase chain reaction (PCR) quantification: TLR4, IL8, NRAMP1, IFNγ and IgY genes in two case and control assays. The 2^−∆∆Ct^ Livak and Schmittgen method was used to compare the expression of genes in treated and untreated samples. This method normalizes the expression of the target gene to that of actin, an internal control and estimates the change in expression relative to the untreated control. Internal control was provided by the Beta actin gene. Next, statistically, the postdoc test was used for the evaluation of treatments using SAS version 9.4, and *p* values of 0.05 and 0.01 were chosen for significant level.

**Results:**

Interestingly, the results of our study suggest the involvement of various factors in the host immune response to *Salmonella*. These include inducible nitric oxide synthase, *NRAMP1*, immunoglobulin light chain (*IgL*), transforming growth factor B family (*TGFb2, TGFb3* and *TGFb4*), interleukin 2 (*IL2*), apoptosis inhibitor protein 1 *(IAP1*), *TLR4*, myeloid differentiation protein 2 (*MD2*), *IFNγ*, caspase 1 (*CASP1*), lipopolysaccharide‐induced tumour necrosis factor (*LITAF*), cluster of differentiation 28 (*CD28*) and prosaposin (*PSAP*). The summary of gene ontology and related genes found for SE resistance was surprisingly comprehensive and covered the following topics: positive regulation of endopeptidase activity, interleukin‐8 production, chemokine production, interferon‐gamma production, interleukin‐6 production, positive regulation of mononuclear cell proliferation and response to interferon‐gamma. The role of these promising biomarkers in our networks against SE susceptibility is essentially confirmed by these results. After 48 h, the spleen showed significant expression of the tissue‐specific gene expression patterns for *NRAMP1* and *IL8* in the cecum, spleen and liver. Based on this information, this report searches for resistance and susceptibility lineages in most genomic regions for SE.

**Conclusions:**

In conclusion, the development of an appropriate selection program to improve resistance to salmonellosis can be facilitated by a comprehensive understanding of the immune responses of the chicken immune system after SE exposure.

## Graphical abstract

This research specifically highlights the importance of avian host immune system against *Salmonella* and double confirmed role of candidate genes expression in response to SE challenges.

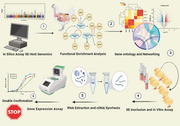

## 1 INTRODUCTION

*Salmonella enteritidis* (SE), a previously widespread infectious disease, is still cited as a major factor in economic losses in commercial chicken production (Sevilla‐Navarro et al., 2020; Zhang et al., 2021). The host's genetic immune system determines the pathogenicity of a particular bacterium (Ehrlich et al., 2008).

Several strategies have been proposed to prevent salmonellosis (Mkangara, 2023). Chicken can often be protected from SE infections through vaccinations, good hygiene and the use of antibiotics. By selecting more resistant animals, we can increase genetic resistance in the fight against salmonellosis (Calenge et al., 2010). One category of threshold traits is resistance to SE. A large number of genes, each with a relatively small influence, combine to shape the architecture of these traits. Next, acquiring information is typically risky, inconvenient and not heritable. Resistance to SE is not strongly inherited. Therefore, there are numerous practical difficulties associated with direct selection for disease resistance. The range of estimated heritability for resistance to salmonellosis was 0.06–0.26 (Berthelot et al., 1998; Calenge et al., 2010; Janss & Bolder, 2000).

For poultry geneticists, breeding to improve disease resistance is the biggest challenge to overcome. Strengthening an animal's resistance to infectious disease has several benefits, such as an improvement in animal welfare, an increase in productivity and efficiency as well as a smaller ecological footprint and a reduced need for additional disease control measures and increased public awareness (Detilleux, 2001; Düpjan & Dawkins, 2022; Knap & Doeschl‐Wilson, 2020). However, breeding for disease resistance presents many challenges (Bai & Plastow, 2022).

In silico studies play a pivotal role in understanding and combating disease resistance. These computational simulations and analyses offer a powerful tool for researchers to explore and predict the behaviour of pathogens, host organisms and their interactions. By utilizing in silico studies, researchers can unravel the underlying mechanisms of disease resistance and identify potential drug targets. Furthermore, these simulations enable the testing of various scenarios and hypotheses, providing valuable insights into the dynamics of diseases and the effectiveness of potential treatments.

RNA sequencing (RNA‐Seq) helps to reveal host genetic mechanisms for disease resistance and to identify candidate genes. RNA‐Seq of indigenous and commercial chickens infected with *Salmonella typhimurium* (ST) showed the difference in expression of 3153 and 1787 genes in liver and spleen, respectively. Stimulator of interferon genes (search tool for the retrieval of interacting genes/protein [STRING]) database (http://string‐db.org/) was used to analyse the protein–protein interaction network (*PPI*) and Cytoscape software v3.8.2 was used to draw the gene network (Dar et al., 2022). In the study by Wang et al. (2023) in addition to gene expression analysis by RNA‐Seq, the co‐expression of genes and the association of gene expression with changes in immune traits, as well as the dynamics of temporary gene expression changes in chickens infected with SE, were investigated using the weighted gene co‐expression network analysis.

The strengths of bioinformatics tools in the study of biomolecules, such as peptides, are the speed of the software despite the huge data, simple use, and improvement of the accuracy of the outputs (Rivero‐Pino et al., 2023). Although in silico study tools offer numerous advantages, they do have certain limitations. One major limitation is the accuracy of the models used in simulations. The accuracy of predictions heavily relies on the quality of the input data and the assumptions made in the model. Inaccurate or incomplete data can lead to unreliable results. Another limitation is the lack of experimental validation (Bhat et al., 2022; Chang et al., 2022; Marbach et al., 2010). Despite these limitations, in silico studies remain a valuable tool in disease resistance research, providing valuable insights and guiding experimental investigations.

Due to their important roles in immune functions, numerous genes representing potential lines of resistance and susceptibility to SE have recently been found (Calenge et al., 2010, Tohidi et al., 2018). Therefore, this work aims to identify the most important immune‐related genes along with their signalling pathways (gene ontology, co‐expression and interactions), accumulation analysis and host‐resistant SE diseases. This motivation led to the main goal of the current review, namely, to investigate how network and systems biology approaches can be used to study the gene regulatory interactions between SE and its host in chickens. To identify the key immune‐related genes, their signalling pathways (gene ontology, co‐expression and interactions) and accumulation analyses for host‐resistant SE diseases, this work combines model‐based in silico evidence with gene expression assays.

## 2 MATERIALS AND METHODS

### 2.1 In silico networking of SE‐host genomics: initial material for interpretation

Systematic reviews are a valuable tool for acquiring concepts, identifying gaps in knowledge and prioritizing areas for future research in chicken genomics research on SE. For this reason, the Preferred Reporting Items for Systematic reviews and Meta‐Analyses protocol was used to conduct a systematic review of significant candidate genes affecting SE (Woods et al., 2006). A total of 40 journal articles were collected and studied for the primary study, and those with low validity were not included in the subsequent analysis. Search engines, Science Direct, PubMed, SCIRUS, Google Scholar, Oxford Journals, Cambridge Journals, Springer Journals, Wiley Library and the NCBI website were the sources of new material for this report. *Salmonella*, candidate gene, quantitative trait locus (QTL) mapping, gene expression, connectivity and gene ontology, genome‐wide association studies and RNA‐Seq profiles were the most frequently selected keywords to search for the target papers.

The majority of the papers were written in English, and the search was strictly limited to the *Gallus gallus* species. Paper sorting and deduplication were done using Endnote software. In summary, results from text‐based surveys and online computational tools from Phenomenon (https://genemania.org) have been translated to a significant statistical level for *Salmonella*‐associated genomic regions and their network pathways using in silico models in sheep, and the reader is provided with a short list of the most important genes involved in SE. Criteria for connectivity assessed were inter‐centrality, properties, co‐expression, expected genetic interactions, signalling pathways, physical interactions, shared protein domains and co‐locations. Table 1 indicates a snapshot of the gene list associated with the immune system in *G. gallus* species genome.

**TABLE 1:** Snapshot of gene list associated with immune system in *Gallus gallus* species genome.

Chr.	Address	Start	End	Gene name	Full name
1	NC_006088.5	19333596	19338292	PIM3	Proto‐oncogene, serine/threonine kinase
1	NC_006088.5	197467876	197473316	ILK	Integrin‐linked protein kinase
1	NC_006093.5	12653093	12673291	PSAP	Prosaposin
1	NC_006091.5	53716432	53719895	IL2	Interleukin 2
1	NC_006095.5	21501344	21507655	PRDX1	Peroxiredoxin‐1
1	NC_006088.5	119778662	119787313	PRDX4	Peroxiredoxin 4
1	NC_006088.5	38477995	39006643	NAV3	Neuron navigator 3
2	NC_006094.5	22184822	22193860	PTPRN	Protein Tyrosine Phosphatase Receptor Type N
2	NC_006102.5	11550591	11610255	NOS1	Nitric oxide synthases
2	NC_006089.5	19795144	19802856	VIM	Vimentin
2	NC_006101.5	12871028	12889227	TRAP1	TNFR‐ASSOCIATED PROTEIN 1 HEAT‐SHOCK PROTEIN
3	NC_006090.5	45355722	45445266	MAP3K4	Mitogen‐activated protein kinase 4
3	NC_006102.5	8213816	8231115	IGLL1	Immunoglobulin lambda like polypeptide 1
4	NC_006088.5	35173603	35177751	IFNG	Interferon gamma
4	NC_006102.5	9578708	9581330	TRIAP1	TNFR‐ASSOCIATED PROTEIN 1 HEAT‐SHOCK PROTEIN
4	NC_006091.5	9588621	9595192	TRAIL‐LIKE	Tumour necrosis factor ligand superfamily member
4	NC_006127.5	56417202	56440160	LMNB1	Laming B1 protein
5	NC_006092.5	29074779	29091844	ARG2	Arginase 2
5	NC_006092.5	29091797	29099825	VTI1B	Vesicle transport through interaction with t‐SNAREs 1B
5	NC_006127.5	899416	904533	SEC11C	EC11 homologue C, signal peptidase complex subunit
5	NC_006094.5	22547346	22552954	SLC11A1	Solute carrier family 11 member 1
6	NC_006093.5	35730035	35755716	PPP2R2D	Protein tyrosine phosphatase receptor type N
6	NC_006092.5	38669273	38683218	TGFB4	Transforming growth factor, beta‐4
7	NC_006094.5	22223872	22232130	ATG9A	Autophagy‐related protein 9A
7	NC_006092.5	12678796	12686490	SAAL1	Serum amyloid a like 1
8	NC_006095.5	24152953	24295132	FAF1	FAS‐associated factor
8	NC_006090.5	19449226	19512091	TGFB2	Transforming growth factor, beta‐2
14	NC_006101.5	6375555	6377491	GNG13	Guanine nucleotide–binding protein G(I)/G(S)/G(O) subunit gamma‐13
14	NC_006104.5	3939009	3944463	TLR4	Toll‐like receptor 4
14	NC_006089.5	64356480	64377248	TXNDC5	Thioredoxin domain–containing protein 5
14	NC_006091.5	53716432	53719895	IL2	Interleukin 2
15	NC_006106.5	844666	849704	CASP1	Cysteine‐aspartic acid protease (caspase)
15	NC_006088.5	35173603	35177751	IFNG	Interferon gamma
15	NC_006101.5	4434280	4445522	MMD2	Monocyte to macrophage differentiation associated 2
15	NC_006102.5	8213816	8231115	IGLL1	Immunoglobulin Lambda Like Polypeptide 1
16	NC_006103.5	2601161	2604724	TAP2	Transporter 2, ATP binding cassette subfamily B member
16	NC_006103.5	2595947	2601142	TAP1	Transporter 2, ATP binding cassette subfamily B member
16	NC_006103.5	2454628	2458853	TRIM7	Tripartite motif containing 7
16	NC_006103.5	2473440	2478234	IL4I1	Interleukin 4 induced 1
16	NC_006103.5	2500960	2511817	TRIM27	Tripartite motif containing 27
17	NC_006101.5	1329345	1356141	LITAF	Lipopolysaccharide‐induced TNF facto
18	NC_006105.5	5812317	5833003	TOM1L1	Target of Myb1 Like 1 Membrane Trafficking **Protein**
18	NC_006105.5	5906619	5940016	HLF	Hepatic leukemia factor
18	NC_006105.5	5945777	5989535	MMD	Monocyte to macrophage differentiation factor
19	NC_006106.5	9280866	9338537	KSR1	Kinase Suppressor Of Ras 1
19	NC_006106.5	9451318	9463360	TNFAIP1	TNF alpha induced protein 1
19	NC_006106.5	9465671	9510155	SGSM2	Small G protein signalling modulator 2
19	NC_006106.5	9585934	9615086	PAFAH1B1	Platelet activating factor acetylhydrolase 1b regulatory subunit 1
19	NC_006089.5	116735494	116749578	TRAM1	Translocation associated membrane protein 1
23	NC_006110.5	1832023	1846204	ZMPSTE24	Zinc metallopeptidase STE24
23	NC_006110.5	1880370	1886751	C2	Complement **C2**
23	NC_006110.5	1905026	1905821	SFN	Stratifin is a protein
27	NC_006114.5	5745953	5749615	SLC35B1	Solute carrier family 35 member B1
Z	NC_006088.5	63277207	63283946	MGST1	Microsomal glutathione S‐transferase 1
Z	NC_006091.5	11329904	11334359	IRG1L	Immunoresponsive gene 1, like

### 2.2 Functional enrichment analysis

To gain further insights into the highlighted candidate gene, biological terms and pathways, functional enrichment analysis was performed. For this purpose, the *DAVID* version 6.8 (Annotation, Visualization and Integrated Discovery) database (Sherman et al., 2022) was applied using standard parameters, focusing on the Gene Ontology (*GO*; biological process) and the Kyoto Encyclopedia of Genes and Genomes (*KEGG*) gene ontology terms and pathways enriched with *FDR *< 0.05 were considered significant. Table 2 outputs of in silico modelling and networking associated with *Salmonella*‐host genomics.

**TABLE 2:** Outputs of in silico modelling and networking associated with *Salmonella*‐host genomics.

No	Candidate gene	Degree	Betweenness	Closeness
1	TLR4	5.0	154.0	0.056798622
2	PRDX1	4.0	161.0	0.056798622
3	SLC11A1	4.0	84.0	0.056027167
4	IFNG	4.0	10.0	0.055932205
5	SLC35B1	3.0	42.0	0.055743244
6	CASP1	3.0	0.0	0.055743244
7	SEC11C	3.0	6.0	0.054908484
8	IL2	3.0	32.0	0.055555556
9	MGST1	3.0	70.0	0.055932205
10	TGFB2	3.0	62.0	0.054908484
11	TXNDC5	3.0	20.0	0.05564924
12	PRDX4	3.0	9.0	0.05564924
13	PSAP	2.0	32.0	0.054635763
12	TRIM7	1.0	0.0	0.030303031
15	PTPRN	1.0	0.0	0.030303031
16	MMD	1.0	0.0	0.030303031
17	TOM1L1	1.0	0.0	0.030303031
18	VIM	1.0	0.0	0.053484604
19	SGSM2	1.0	0.0	0.030303031
20	PAFAH1B1	1.0	0.0	0.030303031
21	LITAF	1.0	0.0	0.05409836
21	TNFAIP1	1.0	0.0	0.030303031
23	FAF1	1.0	0.0	0.030303031
24	ZMPSTE24	1.0	0.0	0.030303031
25	LMNB1	1.0	0.0	0.030303031
26	NAV3	1.0	0.0	0.054276317
27	GNG13	1.0	0.0	0.053225808
28	IGLL1	1.0	0.0	0.030303031
29	C2	1.0	0.0	0.030303031
30	VTI1B	1.0	0.0	0.053484604
31	TAP2	1.0	0.0	0.030303031
32	TAP1	1.0	0.0	0.030303031
33	NOS1	1.0	0.0	0.030303031
34	ARG2	1.0	0.0	0.030303031

### 2.3 Network analysis

To uncover the *PPIs* between the genes of the *KEGG* pathway, the STRING database (the Search Tool for the Retrieval of Interacting Genes/Proteins) (version 11.5) was applied using a chicken reference organism. The experimentally validated text mining, database and co‐expression interactions were taken into account for network construction. To exclude unreliable *PPIs*, a confidence value < 0.4 (a commonly used threshold) was adopted. In addition, the *PPI* networks were clustered into a certain number of clusters (three clusters) in the database based on the *k*‐means clustering approach.

### 2.4 Validation assay for identified promising biomarkers

Forty birds, including village chickens and red jungle fowl, were used to examine the degree of previously identified candidate gene expression folding within 48 h of vaccination. Day‐old chicks were randomly assigned to wire‐bottomed cages and placed in a biosafety level 2 experimental room. Twenty of the wing‐marked chicks received an injection of 1 × 10^7^ CFU/mL of SE type 13a phage. The remaining chicks received a 0.1 mL mock inoculation with Luria‐Bertani broth. SE phage type 13a was cultured in XLD agar (Oxiod) and incubated at 37°C for 24 h. The inoculum was prepared from the second subculture of the bacteria. The dose of 1 × 10^8^ CFU/mL of SE inoculation was determined using the McFarland standard. The day old chicks were inoculated with 1 × 10^7^ CFU/mL SE (0.1 mL of inoculums) intraesophageal by a syringe provided with infusion teat as described.

Two and forty‐eight hours after inoculation, the chicks were sacrificed by cervical dislocation. The liver, spleen and caecum of each chick were surgically removed and then cleaned with phosphate‐buffered saline. The samples were placed in liquid nitrogen and immediately covered with aluminium foil. Samples were then kept cold at −80°C until RNA was extracted. Following the manufacturer's instructions, RNA was extracted from less than 30 mg of tissue using the RNeasy Mini Kit (QIAGEN).

Total RNA was converted to complementary DNA (cDNA) using the QuantiTect Reverse Transcription Kit (QIAGEN) and the manufacturer's protocol. Using a Quantify Corbett 6000 real‐time PCR machine, a fold change in the expression of Toll‐like receptor 4 (*TLR4*), interleukin 8 (*IL8)*, interferon g *(IFNg)*, natural resistance‐associated macrophage protein 1 (*NRAMP1)* and immunoglobulin Y (*IgY*) genes was detected in village chicken and jungle fowl chicken in the first 2 and 42 h after inoculation SE. These five genes are involved in innate and acquired immune responses. Previous studies revealed their activity in response to SE and *Salmonella Typhimurium* (Hu et al., 1997; Kogut, 2002; Kogut et al., 2005; Li et al., 2017). In the PCR reaction, 12.5 µL Maxima SYBR Green qPCR master mix, 3 mL of each primer (Table 3), 100 ng cDNA and nuclease‐free water were used, which had a total volume of 25 µL. Table 3 addresses primer pairs used for chicken Messenger RNA (mRNA) quantification for five promising highlighted candidate genes by real‐time PCR.

**TABLE 3:** Primer pairs used for chicken mRNA quantification by real‐time PCR.

Gene	Primer	Product size	Annealing temperature (°C)	Accession number
*TLR4*	F: 5′AGA GCA AAG ACC TAA ATG GC3′ R: 5′GGT AAG GAA GGA GAG ACG ATT TC3′	95	60	NM_001030693.1
*IL8*	F: 5′AGATCCCTTGGAAGCCACTT R: 5′GGAATTACCAGTTTGCTGCTG3′	118	60	NM_205498.1
*NRAMP1*	F: 5′CTACGAGTATGTGATGGTGC3′ R: 5′GTGCAGGAAGATGTTATGGG3′	148	60	NM_204964.1
*IFNγ*	F: 5′GGA AGT AGA CTT GCT TTA AGG TGAG3′ R: 5′CAA TAG AGT GTC ACA TTT CCT GGAG3′	150	60	NM_205149.1
*IgY*	F: 5′CAGCTTAGACGCCAAACTG3′ R: 5′TGTAGGTGCCGTTGAAGTG3′	139	55	X07176.1
*β actin*	F: 5′CGC ATA AAA CAA GAC GAG ATTGG3′ R: 5′GGCGTTCGCTCCAACATG3′	132	55/60	NM_205518

Abbreviations: mRNA, Messenger RNA; PCR, polymerase chain reaction.

The amplification cycle was set up with an initial denaturation time of 10 min at 95°C, 40 cycles of 1 min at 95°C and 60 s at 60°C for the annealing and extension temperatures. The annealing temperature was set to 55°C for IgY. The temperature increases from 55°C for IgY and 60°C for the remaining genes to 96°C upon completion of the amplification cycle, increasing by 1°C with each cycle to produce a melting curve. Real‐time PCR at 10‐fold dilution of mixed samples was used to determine the efficiency of the reaction for each gene. Only primer sets with efficiencies between 98% and 110% were used.

The 2^−∆∆Ct^ method was used to compare the expression of genes in treated and untreated samples. This method normalizes the expression of the target gene to that of actin, an internal control and estimates the change in expression relative to the untreated control. First, the Ct value for the treated samples and the calibrator or the untreated sample was calculated: ΔCt treated = Ct target treated—Ct reference treated and ΔCt calibrator = Ct target calibrator—Ct reference calibrator.

Then a ΔΔCt value for each of the treated samples was calculated: ΔΔCt treated sample = ΔCt treated sample—ΔCt calibrator sample. Finally, relate the target gene expression in each of the treated samples to that in the calibrator sample: change in gene expression = 2^−ΔΔCt^. This is relative quantity. The fold change was expressed as their log 10 scale to reach to normal distribution. Statistically, the post hoc test was used for the evaluation of treatments using SAS version 9.4 and *p* value of 0.05 and 0.01 was chosen for significant levels.

## 3 RESULTS

### 3.1 In silico networking of SE‐host genomics: initial material for interpretation

**FIGURE 1:**
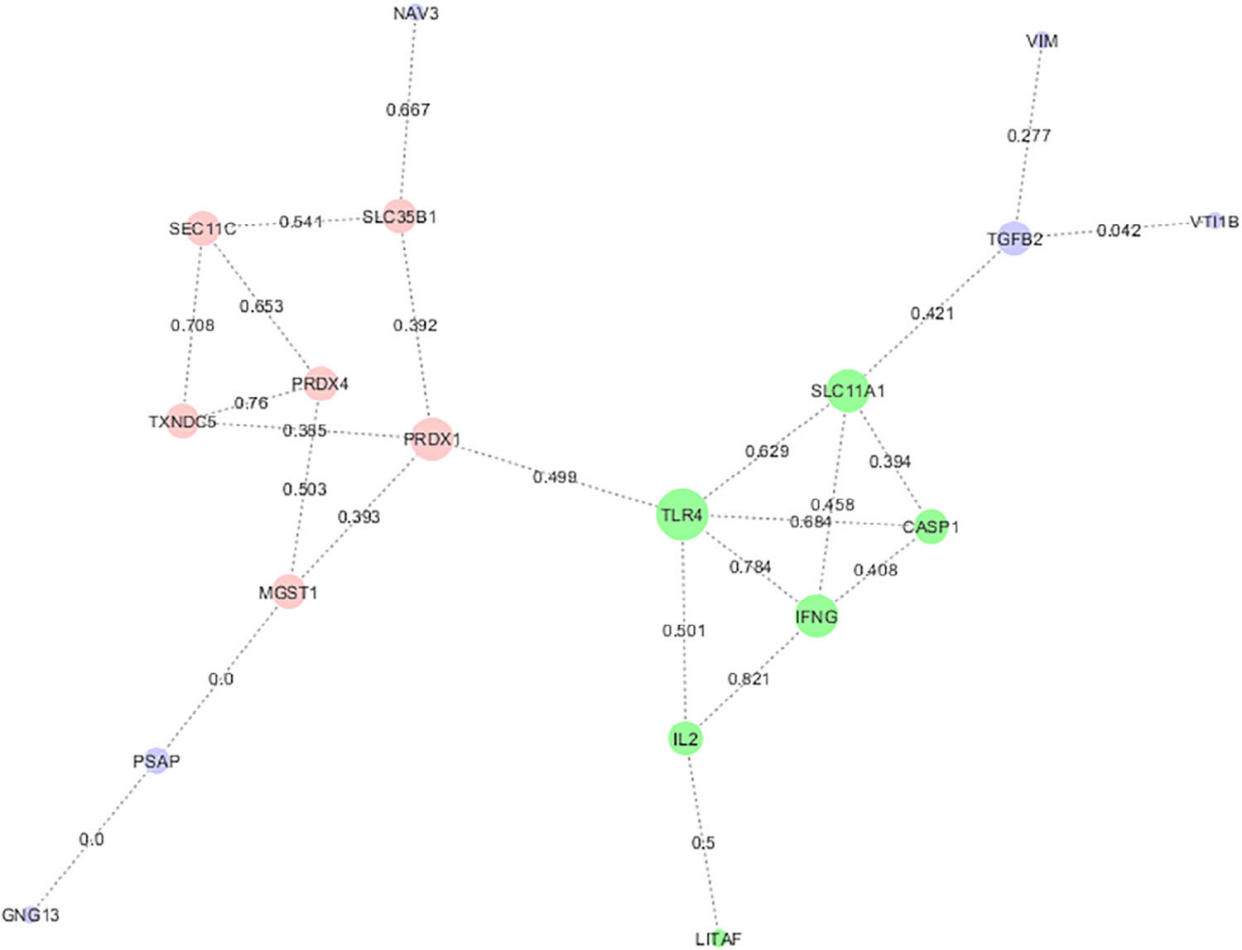
Significant key candidate gene based in silico evidence against *Salmonella enteritidis* (SE) in chicken.

The results of our study are noteworthy as they suggest the involvement of various factors in the host immune response to *Salmonella*. These include inducible nitric oxide synthase (iNOS), *NRAMP1*, immunoglobulin light chain (*IgL*), transforming growth factor B family (*TGFb2, TGFb3* and *TGFb4*), interleukin 2 (IL2), apoptosis inhibitor protein 1 (*IAP1*), *TLR4*, myeloid differentiation protein 2 (*MD2*), *IFNg*, caspase 1 (*CASP1*), lipopolysaccharide‐induced tumour necrosis factor (TNF) factor (*LITAF*), cluster of differentiation 28 (*CD28*) and prosaposin (*PSAP*). Figure 1 illustrates significant key candidate gene‐based in silico evidence against SE in chicken.

### 3.2 Functional enrichment analysis

**FIGURE 2:**
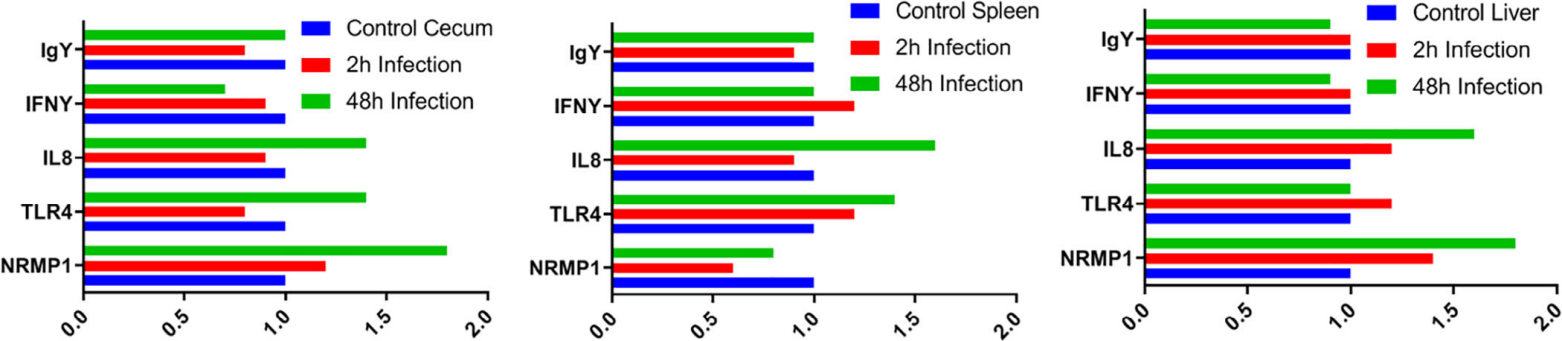
Outputs of relative gene expression quantification in three tissues and two promising biomarkers in response to *Salmonella enteritidis* (SE) inoculation after 2 and 48 h infection significant biological pathways.

To gain further insights into the functional enrichment analysis, the summary of gene ontology and related genes found for SE resistance was surprisingly comprehensive and covered the following topics: positive regulation of endopeptidase activity, interleukin‐8 production, chemokine production, interferon‐gamma production, interleukin‐6 production, interleukin‐8 production, positive regulation of mononuclear cell proliferation and response to interferon‐gamma. Table 4 shows a summary of gene ontology and associated gene found for SE resistance. Table 5 illustrates a summary of literature review on gene networking for comparison with present outcomes against SE. Figure 2 illustrates outputs of relative gene expression quantification in three tissue and two promising biomarkers in response to SE inoculation after 2 and 48 h infection significant biological pathways.

**TABLE 4:** Summary of gene ontology and associated gene found for *Salmonella enteritidis* (SE) resistance.

GOID	GOTerm	Associated Genes Found
GO:0009615	Response to virus	[EIF2AK2, FADD, IFIT5, MAPK11, MYD88, OASL and PARP9]
GO:0006919	Activation of cysteine‐type endopeptidase activity involved in apoptotic process	[APAF1, CASP1 and NOD1]
GO:0032637	Interleukin‐8 production	[FADD, NOD1 and TLR4]
GO:0043122	Regulation of I‐kappaB kinase/NF‐kappaB signalling	[FADD, MYD88, NOD1, TLR4 and TNFSF10]
GO:0043123	Positive regulation of I‐kappaB kinase/NF‐kappaB signalling	[FADD, MYD88, NOD1, TLR4 and TNFSF10]
GO:0010952	Positive regulation of peptidase activity	[APAF1, CASP1, FADD, NOD1 and TNFSF10]
GO:0097153	Cysteine‐type endopeptidase activity involved in apoptotic process	[APAF1, BIRC3, CASP1, NOD1 and TNFSF10]
GO:0010950	Positive regulation of endopeptidase activity	[APAF1, CASP1, FADD, NOD1 and TNFSF10]
GO:0043281	Regulation of cysteine‐type endopeptidase activity involved in apoptotic process	[APAF1, BIRC3, CASP1, NOD1 and TNFSF10]
GO:2000116	Regulation of cysteine‐type endopeptidase activity	[APAF1, BIRC3, CASP1, FADD, NOD1 and TNFSF10]
GO:0043280	Positive regulation of cysteine‐type endopeptidase activity involved in apoptotic process	[APAF1, CASP1, NOD1 and TNFSF10]
GO:2001056	Positive regulation of cysteine‐type endopeptidase activity	[APAF1, CASP1, FADD, NOD1 and TNFSF10]
GO:0032602	Chemokine production	[EIF2AK2, MYD88 and TLR4]
GO:0032609	Interferon‐gamma production	[EOMES, FADD, PDE4D, SLC11A1 and TLR4]
GO:0032635	Interleukin‐6 production	[MYD88, NOD1 and TLR4]
GO:0032637	Interleukin‐8 production	[FADD, NOD1 and TLR4]
GO:0071216	Cellular response to biotic stimulus	[IL8L1, LITAF, MYD88, PDE4D and TLR4]
GO:0071706	Tumour necrosis factor superfamily cytokine production	[FADD, MYD88, NOD1 and TLR4]
GO:0001819	Positive regulation of cytokine production	[EIF2AK2, FADD, FST, MAPK11, MYD88, NOD1, PDE4D, SLC11A1 and TLR4]
GO:0032640	Tumour necrosis factor production	[FADD, MYD88, NOD1 and TLR4]
GO:0060759	Regulation of response to cytokine stimulus	[FADD, PARP9 and TLR4]
GO:0032642	Regulation of chemokine production	[EIF2AK2, MYD88 and TLR4]
GO:0032649	Regulation of interferon‐gamma production	[FADD, PDE4D, SLC11A1 and TLR4]
GO:0032675	Regulation of interleukin‐6 production	[MYD88, NOD1 and TLR4]
GO:0042107	Cytokine metabolic process	[MYD88, NOD1 and TLR4]
GO:0050918	Positive chemotaxis	[AvBD1, AvBD7, CXCL12, DEFB4A and VEGFA]
GO:0060326	Cell chemotaxis	[AvBD1, AvBD7, CCL26, CXCL12, DEFB4A, IL8L1, PDE4D and VEGFA]
GO:0060760	Positive regulation of response to cytokine stimulus	[FADD, PARP9 and TLR4]
GO:0070665	Positive regulation of leukocyte proliferation	[FADD, IL2, MYD88 and TLR4]
GO:1903555	Regulation of tumour necrosis factor superfamily cytokine production	[FADD, MYD88, NOD1 and TLR4]
GO:0072676	Lymphocyte migration	[CCL26, FADD and IL8L1]
GO:0097529	Myeloid leukocyte migration	[CCL26, IL8L1, MYD88, PDE4D and VEGFA]
GO:0002237	Response to molecule of bacterial origin	[IL8L1, LITAF, MYD88, PDE4D, SLC11A1 and TLR4]
GO:0032722	Positive regulation of chemokine production	[EIF2AK2, MYD88 and TLR4]
GO:0032729	Positive regulation of interferon‐gamma production	[FADD, PDE4D, SLC11A1 and TLR4]
GO:0032755	Positive regulation of interleukin‐6 production	[MYD88, NOD1 and TLR4]
GO:0038061	NIK/NF‐kappaB signalling	[EIF2AK2, LITAF, MYD88, NOD1 and TLR4]
GO:0042742	Defense response to bacterium	[AvBD1, AvBD7, DEFB4A, MYD88, NOD1, SLC11A1 and TLR4]
GO:1903557	Positive regulation of tumour necrosis factor superfamily cytokine production	[FADD, MYD88, NOD1 and TLR4]
GO:0032496	Response to lipopolysaccharide	[IL8L1, LITAF, MYD88, PDE4D, SLC11A1 and TLR4]
GO:0071219	Cellular response to molecule of bacterial origin	[IL8L1, LITAF, MYD88, PDE4D and TLR4]
GO:0005125	Cytokine activity	[CCL26, CXCL12, IL2, IL8L1, LITAF, TGFB1 and TNFSF10]
GO:0032680	Regulation of tumour necrosis factor production	[FADD, MYD88, NOD1 and TLR4]
GO:0032946	Positive regulation of mononuclear cell proliferation	[FADD, IL2, MYD88 and TLR4]
GO:0034341	Response to interferon‐gamma	[CCL26, PARP9, SLC11A1 and TLR4]
GO:0042056	Chemoattractant activity	[AvBD1, AvBD7, DEFB4A and VEGFA]
GO:0050729	Positive regulation of inflammatory response	[IL8L1, MYD88 and TLR4]
GO:0097530	Granulocyte migration	[CCL26, IL8L1, MYD88 and PDE4D]
GO:0032760	Positive regulation of tumour necrosis factor production	[FADD, MYD88, NOD1 and TLR4]
GO:0043122	Regulation of I‐kappaB kinase/NF‐kappaB signalling	[FADD, MYD88, NOD1, TLR4 and TNFSF10]
GO:0050829	Defense response to Gram‐negative bacterium	[AvBD7, SLC11A1 and TLR4]
GO:0070098	Chemokine‐mediated signalling pathway	[CCL26, CXCL12 and IL8L1]
GO:0071621	Granulocyte chemotaxis	[CCL26, IL8L1 and PDE4D]
GO:1901222	Regulation of NIK/NF‐kappaB signalling	[EIF2AK2, LITAF, MYD88, NOD1 and TLR4]
GO:0071222	Cellular response to lipopolysaccharide	[IL8L1, LITAF, MYD88, PDE4D and TLR4]
GO:0008009	Chemokine activity	[CCL26, CXCL12 and IL8L1]
GO:0043123	Positive regulation of I‐kappaB kinase/NF‐kappaB signalling	[FADD, MYD88, NOD1, TLR4 and TNFSF10]
GO:0050671	Positive regulation of lymphocyte proliferation	[FADD, IL2, MYD88 and TLR4]
GO:0070304	Positive regulation of stress‐activated protein kinase signalling cascade	[EIF2AK2, MYD88, NOD1 and TLR4]
GO:0071346	Cellular response to interferon‐gamma	[CCL26, PARP9 and TLR4]
GO:1901224	Positive regulation of NIK/NF‐kappaB signalling	[EIF2AK2, MYD88, NOD1 and TLR4]
GO:1990266	Neutrophil migration	[CCL26, IL8L1, MYD88 and PDE4D]
GO:0030593	Neutrophil chemotaxis	[CCL26, IL8L1 and PDE4D]
GO:0010952	Positive regulation of peptidase activity	[APAF1, CASP1, FADD, NOD1 and TNFSF10]
GO:0010950	Positive regulation of endopeptidase activity	[APAF1, CASP1, FADD, NOD1 and TNFSF10]
GO:2000116	Regulation of cysteine‐type endopeptidase activity	[APAF1, BIRC3, CASP1, FADD, NOD1 and TNFSF10]
GO:0051092	Positive regulation of NF‐kappaB transcription factor activity	[EIF2AK2, MYD88, NOD1 and TLR4]
GO:0032874	Positive regulation of stress‐activated MAPK cascade	[EIF2AK2, MYD88, NOD1 and TLR4]
GO:2001056	Positive regulation of cysteine‐type endopeptidase activity	[APAF1, CASP1, FADD, NOD1 and TNFSF10]

Abbreviation: CXCL, chemokine (C‐X‐C motif) ligand 1.

**TABLE 5:** Summary of literature review on gene networking for comparison with present outcomes against *Salmonella enteritidis* (SE).

Gene pathway and signalling	Gene networks and ID
Toll like receptors	MD2, TLR‐1, TLR‐2, TLR‐4 and TLR‐5
Cytokines	Ah249, IL2, Il4, IL6, IL8, IL10, IL18, IFNG, K60 MIP1B, SOCS3, TGFB2 and TGFB4
Apoptosis	BcI‐X, CASP1, IAP, TRAL and TNF1
Atimicrobial peptides	AVBD2, AVBD3, AVBD5, AVBD11 and AVBD12
Cell surface antigens	CD3, CD40, MHC2A and MHC2B

### 3.3 Network analysis and 3D protein modelling

**FIGURE 3:**
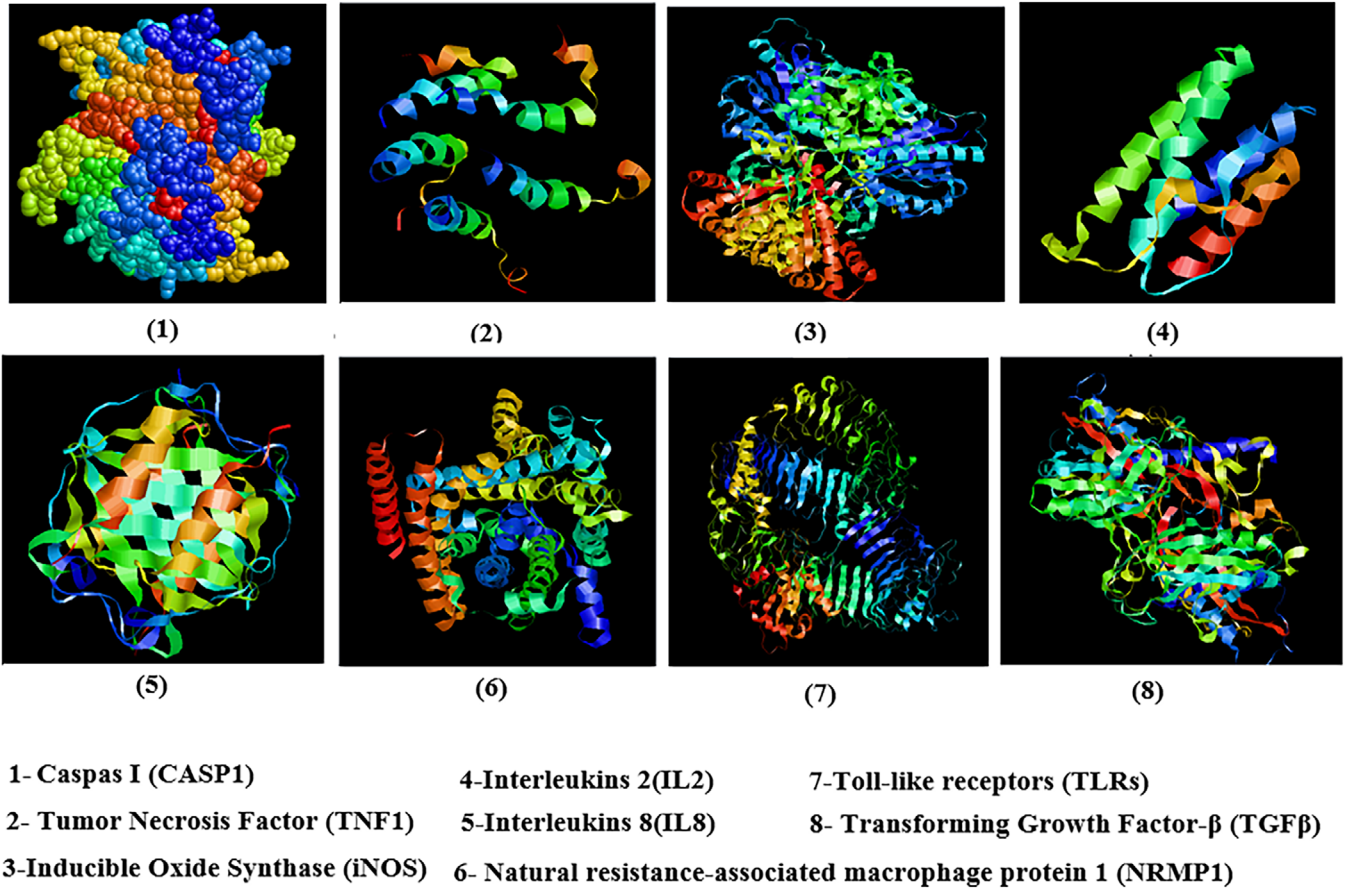
3D protein modelling for significant key candidate gene–based in silico evidence against *Salmonella enteritidis* (SE) in chicken. Host chicken big or small structure of protein of gene somehow not associated with susceptibility or resistance to SE infection and it seems that type of amino acid and 3D dominion of associated protein with host immune system and location of single nucleotide polymorphisms (SNP) play fundamental role for mechanism of up or down regulation of gene and some novel approach also is what is relationship between structure and size of proteins and their gene sequence with place in chromosome number or ordering in certain Chr. length.

To uncover the *PPIs* between the genes of the *KEGG* pathway, the STRING database (the Search Tool for the Retrieval of Interacting Genes/Proteins) (version 11.5) was applied using a chicken reference organism. Figure 3 illustrates output 3D protein gene modelling and predication for identified candidate genes against SE.

### 3.4 Validation assay for identified promising biomarkers

After 48 h, the spleen showed significant expression of the tissue‐specific gene expression patterns for *NRAMP1* and *IL8* in the cecum, spleen and liver. Based on this information, this report searches for resistance and susceptibility lineages in most genomic regions for SE.

## 4 DISCUSSION

The host's genetic immune system determines the pathogenicity of a particular bacterium. To shed light on this topic, it was necessary to understand the key candidate genes essential for regulating susceptibility and resistance to the target disease (Le et al., 2004). The field of poultry farming in particular has benefited greatly from the connection between quantitative and molecular genetics.

### 4.1 In silico networking of SE‐host genomics: initial material for interpretation

Based on the literature on SE susceptibility and desirability, we elucidated the properties and performance of 16 top candidate genes for signatures. Some of the works of literature review for identified genes are as follows.

#### 4.1.1 Interleukin 2 (IL2)

A cytokine that is essential for T‐cell activation and differentiation is *IL2*. The process of *IL2* synthesis occurs when antigens activate *CD4+* T cells, which in turn stimulates transcription of the *IL2* gene (Lan et al., 2008). T cells, B cells and natural killer (NK) cells are the cells that *IL2* targets. The main effects of this protein are the activation and proliferation of these targeted cells (Habib et al., 2003). The mammalian *IL2* gene and the chicken *IL2* gene have a similar genomic structure. The gene has three introns and four exons, but the introns have two, and three are shorter in chicks than in mammals (Kaiser & Mariani, 1999). After exposure to the *Brucella abortus* antigen, the interaction of *IL2* gene polymorphisms with *IFNg* significantly influenced antibody titres in chickens. *Salmonella* Load in the cecum and liver of broilers has been associated with different IL2 genotypes. New insertions and deletions in the *IL2* intron region of domestic and commercial chickens were reported (Zhang et al., 2011). Studying the association of new mutations with resistance to diseases may aid to identify new genetic markers.

#### 4.1.2 Inducible nitric oxide synthase (iNOS)

When *TLRs* are activated in the presence of microbial products, macrophages release the enzyme known as induced NO synthase (Abdul‐Cader et al., 2016). Unstimulated macrophages do not contain iNOS (Xiong et al., 2000). NO diffuses through the release of iNOS, which catalyses the conversion of arginine to citroline. NO can react with superoxide or hydrogen peroxide in phagolysosomes and produce peroxynitrite radicals that can kill bacteria (Madkour, 2019). Chickens are a good animal model to study the expression of the iNOS gene because they cannot regrow and meet their nutritional requirements for this amino acid (Wang et al., 2022). There is 66.6% and 70.4% sequence similarity between the chicken iNOS protein and the mouse and human iNOS, respectively. Similar to human iNOS mRNA, chicken iNOS mRNA is 4.5 kb in size. NF‐kB is involved in the regulation of iNOS expression in chickens (Lin et al., 1996). When *Eimeria tenella* was present in the cecum of chickens, the expression of iNOS increased up to 200‐fold (Hong et al., 2006). Table 6 highlights a literature review on gene expression on resistance and susceptibility to SE.

**TABLE 6:** Literature review on gene expression on resistance and susceptibility to *Salmonella enteritidis* (SE).

Row	Gene names and symbol	Functionality
1	MHC class I	SE colonialization
2	MHC class II	SE colonialization
3	IL‐6	Resistance and susceptibility to SE
4	IL‐8	Resistance and susceptibility to SE
5	IL‐18	Resistance and susceptibility to SE
6	CASP1	Resistance and susceptibility to SE
7	NRMP1	Resistance and susceptibility to SE
8	iNOS	Resistance and susceptibility to SE
9	Complement C	Upregulation in resistance line
10	TRL4	Downregulate in resistance line
11	TGF4B	Downregulate in resistance line
12	IFN	Downregulate in resistance line
13	IL‐2	Resistance and susceptibility to SE
14	TRAL	Resistance and susceptibility to SE
15	Prosaposin	Resistance and susceptibility to SE
16	IAP‐1	Resistance and susceptibility to SE
17	IGL	Resistance and susceptibility to SE
18	SLC11A1	Resistance and susceptibility to SE
19	SAL1	Resistance and susceptibility to SE
20	MYD88	Resistance and susceptibility to SE
21	CD28	Resistance and susceptibility to SE
22	TNF‐alfa	Resistance and susceptibility to SE
23	MD2	Resistance and susceptibility to SE

#### 4.1.3 Toll‐like receptors (TLRs)

*TLRs* are pattern recognition receptors presented on the cell surface. Except for *TLR3*, 7, 8 and 9 that were are located in the membranes of endosomes and lysosomes (Christmas, 2010).

Because they bind to surface ligands of pathogens and release signals, they are crucial for innate immunity. These receptors are expressed primarily on neutrophils, endothelial cells, dendritic cells, mucosal epithelial cells and macrophages (Chang, 2010; Vijay, 2018). Signal transduction occurs in a *Toll/IL1* homology domain (transports internationaux routiers [*TIR*]) in the cytoplasm of the receptor. The *N*‐terminal leucine‐rich repeat domain of *TLR* is located in its extracellular domain (Narayanan & Park, 2015). The specificity of the *TLR* is determined by this section (Li et al., 2017). However, according to Lamont et al. (2008), the *TLR* domain is shared by all *TLRs*. Proinflammatory cytokines, such as *IL‐1*, *IL‐6* and *TNF*, are produced in response to stimulation of *TLR4* that encodes a protein that shares 44% of its similarities with human *TLR4* (Leveque et al., 2003, Degirmenci et al., 2019). Polymorphism in the *TLR4* gene sequence has been shown to affect the survival of chickens infected with ST are infected (Leveque et al., 2003) also in chicken cells.

#### 4.1.4 Caspase 1 (CASP1)

Proteases of the CASP family are necessary for both apoptosis and maturation of cytokines. CASPs 1 and 11 belong to this 14‐member family involved in the proinflammatory cytokine process. By binding to and activating *CASP1*, invasion protein B (Sip B) causes macrophage apoptosis during *Salmonella* infection (Hersh et al., 1999). *CASP1* matures the cytokines *IL1* and *IL18* in this downstream process (Dungan & Mills, 2011). *Salmonella* causes necrosis in infected macrophages through a specific CASP1‐dependent mechanism. *CASP1* triggers the synthesis of *IL1* and *IL18* during necrosis, leading to the release of cytoplasmic contents (Monack et al., 2001). Furthermore, *CASP*3 is not active during necrosis, and DNA is cleaved. However, the maturation of IL1 and IL18 is not required for intracellular clearance induced by *CASP1*. Rather, pyroptic cell death by *CASP1* mediates infection clearance (Lin et al., 2013).

#### 4.1.5 Inhibitor of apoptosis protein 1 (IAP1)

The natural process by which cells die is called apoptosis. Apoptosis occurs in cancer, immunological reactions and homeostasis. *IAP* antagonists limit the action of a family of inhibitory proteins known as IAPs, which inhibit the process of apoptosis. Three components of the apoptotic process are modulated by *IAP, IAP* antagonists and CASP. In two different ways, *IAP* controls CASP to prevent apoptosis (Wei et al., 2008). Both the C‐terminal gene of interest domain (*RING*) and the N‐terminal baculovirus IAP repeat (BIR) are present in every member of the IAP family. With its finger‐like structure, the BIR domain binds to CASPs and blocks their catalytic grooves. According to pervious literatures, the RING domain catalyses the interaction between ubiquitin and CASPs. The sequence of chicken *IAP1*, which has been mapped to chromosome 1, is 85% similar to that of the human IAP gene. In chickens, IAP single nucleotide polymorphisms (SNPs) were associated with the amount of splenic SE and antibody responses in early chicks (Kaiser et al., 1999).

#### 4.1.6 Cluster of differentiation 28 (CD28)

The surface of T cells expresses the membrane protein *CD28* (Yokosuka & Saito, 2009). Transduction signals that activate immature T cells are the main tasks of CD28 (Porciello & Tuosto, 2016). Two signals—one from the interaction of the T‐cell receptor with MHC molecules on antigen‐presenting cells (APCs) and the other from the interaction of co‐stimulatory molecules such as *CD28* on the T‐cell membrane and CD80 and CD86 on the APC membrane—are necessary for T‐cell activation. For *Th2* responses to antigens and the T‐cell proliferation, *CD28* is essential.

#### 4.1.7 Interferon (IFN)

The cytokine interferon serves various immunological purposes in both the innate and adaptive immune systems (Iwasaki & Medzhitov, 2015). When intracellular bacteria are present, *IFN* triggers the activation of macrophages (Snyder et al., 2017). As an immune cell activator, it also has an antiviral function. When activated by *IL12* and *IL18* or by activation of infected cell surface ligands, *NK* cells and *CD4+* and *CD8+* T cells secrete *IFN* (Berg et al., 2002). NO and reactive oxygen are synthesized via IFN. Ten‐day‐old chick embryos expressed IFN in response to RNAs defective in coronavirus. IFN may act as a useful adjuvant to modify subsequent immune responses and enhance the effect of vaccine antigens on immune responses.

#### 4.1.8 Immunoglobulin Y (IgY)

Immunoglobulins (Igs) are the main factors of immune functions in the humoral immune system (Megha & Mohanan, 2021). Immunoglobulins M, A and Y are three classes of avian immunoglobulins, and the latter is a homologue of mammalian I*gG* (Lamont et al., 2008). The chicken *IgY* gene has been mapped to chromosome 15. Early expression of chemokines and *IgM*, *IgA* and *IgY* levels increased in 1‐week‐old chickens infected with ST. The increase in *IgY* and *IgA* levels in chickens infected at 3 and 6 weeks of age was greater than in chickens infected at 1 week of age (Chalghoumi et al., 2009).

### 4.2 Network analysis

To uncover the *PPIs* between the genes of the *KEGG* pathway, the *STRING* database (the Search Tool for the Retrieval of Interacting Genes/Proteins) (version 11.5) was applied using a chicken reference organism.

### 4.3 Validation assay for identified promising biomarkers

Here, based on our research outcomes, the spleen tissue gene expression assay addressed significant expression of the tissue‐specific gene expression patterns for *NRAMP1* and *IL8* in the cecum, spleen and liver.

#### 4.3.1 Natural resistance–associated protein 1 (NRAMP1)

In general, the function of the *NRAMP* family (*NRAMP1* and *NRAMP2*) is the homeostasis of iron and other metals. Previously, *NRAMP1* was found to regulate intracellular macrophages. When pathogens are present, it is produced in the membrane of intracellular vesicles and subsequently transferred to the pathogen's membrane (Searle et al., 1998). By pumping iron ions out of macrophages, *NRAMP1* inhibits the growth of pathogens within macrophages (Qin et al., 2017). The *NRAMP1* gene is a member of a small, highly conserved gene family. The genome sequence of the *NRAMP1* is 1901 bp long. Numerous domains in this protein facilitate the transmission role (Fritsche et al., 2008). According to Malo et al., it was found that mice were more susceptible to infections when aspartic acid modified the glycine at position 169. According to QTL analysis, the susceptibility of mice to SE is related to three loci (Ses1–3). Below the Ses1 locus is the *NRAMP1* gene. In the late stage of the infectious process, it helps in the removal of SE from the reticuleendothelium. NRAMP1 also controls the synthesis of MHC class II, *NO, IL1 and IFNg*. The *NRAMP1* gene is located on chicken chromosome 7q (Hu et al., 1997). Immune traits in chickens have been reported to be associated with *NRAMP1* PCR‐single‐strand conformation polymorphism. PCR, polymerase chain reaction.

*NRAMP2 (Divalent metal transporter 1 [DMT1])* as an iron uptake protein is important in almost all tissues, especially duodenum, liver, thymus and spleen (Forbes & Gros, 2001; He et al., 2013). Moreover, *DMT1* transports divalent cations into the cytosol of cells (Dar et al., 2019). Iron is required, but not essential, for the colonization of SE in the gut of chickens (Wellawa et al., 2022). The upregulation of *DMT1* was observed in chickens infected with ST from days 1 to 9 of infection. The increased expression of DMT1 first occurred in the cecum of ST‐infected chickens, followed by the liver and spleen (Dar et al., 2019).

#### 4.3.2 Interleukin 8 (*IL8*)

*CXCL8*, also known as interleukin 8, is an inflammatory chemokine that promotes the migration of lymphoid cells to the site of inflammation (Russo et al., 2014). Based on their functional role, chemokines can be classified as either inflammatory or homeostatic. According to the distance between the first two cysteines of the chemokine protein, chemokines are divided into four groups: *CXC, CC, CX3C* and C. *IL8* is a member of the *CXC* group. Human and chicken *IL8* map to chromosome 4. The *IL8* gene is conserved between humans and chicks, as evidenced by the intron: exon structure of chicken *IL8* being identical to that of humans. In chickens infected with SE, expression analysis of IL8 revealed an upregulation of this gene 1 week after vaccination (Balestrieri et al., 2008).

## 5 SUMMARY AND CONCLUSION

To our knowledge, our results highlighted a set of known and novel genes/pathways associated with the incidence of SE, using the integration of in silico modelling data with QTL information, which could improve the understanding of molecular mechanisms involved in the incidence of SE in broilers. Resistance to salmonellosis can be increased by developing an appropriate selection program that takes into account the immunological responses of the chicken immune system after SE exposure. The results of our study are intriguing as they suggest the involvement of various factors in the host immune response to *Salmonella*. These include iNOS, *NRAMP1*, *IgL*, *TGFb2, TGFb3* and *TGFb4*, *IL2*, and *IAP1*, *TLR4*, *MD2*, IFNg, *CASP1*, *TNF* Factor (*LITAF*), *CD28* and *PSAP*. The important role of chromosomes 1, 3 and 6 in SE susceptibility and resistance was highlighted in an interesting summary of QTL mapping for SE. After 48 h, the spleen had significantly higher levels of tissue‐specific gene expression patterns for *NRAMP1* and *IL8* compared to the liver, spleen and appendix. This report attempts to identify the lineages responsible for resistance and susceptibility in most genomic regions affected by SE.

## AUTHOR CONTRIBUTIONS

**Reza Tohidi**: Conceptualization; formal analysis. **Hoda Javaheri Bargourooshi**: Writing—review & editing. **Arash Javanmard**: Supervision; data curation; writing—original draft.

## CONFLICT OF INTEREST STATEMENT

The authors declare no conflicts of interest.

### ETHICS STATEMENT

All participants have given their written and informed consent.

### PEER REVIEW

The peer review history for this article is available at https://publons.com/publon/10.1002/vms3.70006.

## Data Availability

The original contributions presented in the study are included in the article; further inquiries can be directed to the corresponding author/s.
